# Diagnostic and therapeutic challenges with malignant biliary tract and periampullary pathology in post-bariatric surgery

**DOI:** 10.3389/fsurg.2026.1833523

**Published:** 2026-07-22

**Authors:** Saksham Gupta, Ishanth Jayewardene, Adeeb Majid

**Affiliations:** Department of General Surgery, John Hunter Hospital, Newcastle, NSW, Australia

**Keywords:** bariatric surgery, endoscopic retrograde cholangiopancreatography, pancreatic cancer, pancreatic surgery, pancreaticoduodenectomy

## Abstract

Patients with prior bariatric surgery present unique challenges when diagnosed with malignant periampullary and biliary tract pathology. Surgically altered gastrointestinal anatomy has significant implications for both diagnostic evaluation and therapeutic intervention, precluding a universal management algorithm. Instead, these patients require an individualized approach to pre-operative assessment, operative planning, and post-operative care. Altered anatomy may influence symptom presentation, limit conventional endoscopic access to the biliary tree, and complicate surgical reconstruction. This review examines the implications of common bariatric procedures on the evaluation and management of periampullary malignancy and proposes a practical framework to guide pancreatic surgeons in the work-up and treatment of this complex patient population.

## 1 Introduction

Malignant biliary tract obstruction in the post bariatric surgical patient is a challenging problem that may require hybrid endoscopic, radiological or surgical techniques in their assessment, and special considerations in individualising resection strategies. Part of the complexity of this process is due to the heterogeneity of bariatric procedures amongst this population. The following review is to assist treating surgeons on an approach to management when encountering such a patient and highlight some of the key factors in decision making.

## 2 Pre-operative

### 2.1 Symptomatology

Bariatric surgical procedures preceding the development of a periampullary malignancy may alter the classic constellation of symptoms extensively described in the oncological literature. Considerations include:

- *Patients after bypass procedures will not present with gastric outlet obstruction***.** As the duodenum is bypassed, these patients will not present with the classical symptoms of vomiting, early satiety and post-prandial fullness that is typically associated with gastric outlet obstruction in the early stages of their disease. This likely contributes to why patients after bariatric surgery are more likely to present with metastatic disease (1). If there is a considerable amount of remnant stomach remaining, there can be some distension of this remnant leading to vague abdominal discomfort, with an occluded gastric remnant having ramifications on the success of any transgastric endoscopic procedures.
- *Weight loss is not a reliable symptom in the 12–18 months after bariatric surgery***.** Pathological weight loss is confounded by therapeutically induced and planned weight loss, though a second phase of weight loss after plateauing should concern the clinician for a malignancy.
- *Diabetic control is affected by both weight loss and pancreatic malignancy***.** Bariatric surgery has been shown to improve diabetic control (2). However, this effect is variable and not observed universally, and may be attenuated by weight regain (3). The development of worsening control despite weight loss from surgery may indicate the development of new pancreatic malignancy

As bariatric procedures do not involve the biliary tract, biliary obstruction leading to jaundice and cholangitis will occur in a similar nature as those without having had bariatric surgery.

### 2.2 Nutrition

At the time of diagnosis, a large proportion of patients with pancreatic cancer will have a compromised nutritional status with significant weight loss. While some patients post bariatric surgery may still be obese, these individuals can still harbour nutritional and vitamin deficiencies, exacerbated further by biliary obstruction. To stimulate greater weight loss through malabsorption, some bariatric procedures create longer biliopancreatic limbs, making nutritional deficiencies for these patients more difficult to correct. Nutrition is key with achieving good perioperative outcomes, and consultation with a dietician experienced with bariatrics can be helpful both pre- and post-operatively. These patients often requirement of replacement of fat-soluble vitamins and pancreatic enzymes. Those who have had a gastric band placed should have their band deflated.

### 2.3 Understanding surgical history

The treating surgeon must obtain the previous surgical record of the bariatric procedures performed on the patient as this information will guide further diagnostic and therapeutic interventions.

Bariatric procedures can be broadly categorised into restrictive and malabsorptive techniques. Restrictive procedures include sleeve gastrectomy, adjustable gastric banding, and vertical gastroplasty. As gastrointestinal continuity is preserved, these operations generally permit conventional endoscopic access to the duodenum and periampullary region, allowing standard gastroscopy and endoscopic retrograde cholangiopancreatography (ERCP) for both diagnostic and therapeutic purposes. Nevertheless, these bariatric procedures still present distinct anatomical and technical considerations at the time of pancreatic or periampullary resection.

Bypass procedures induce weight loss through combined gastric restriction and intestinal malabsorption, achieved by creation of a gastric pouch and diversion of variable lengths of small intestine, with consistent exclusion of the duodenum and proximal jejunum. Roux-en-Y gastric bypass is the archetypal procedure, alongside variants such as single-anastomosis gastric bypass, biliopancreatic diversion with duodenal switch, and single-anastomosis duodeno-ileal (SADI) bypass. The distinct anatomical configurations of these operations necessitate an individualised approach to endoscopic access, and operative approach.

The key factors the treating team must find out include:

- The type of bariatric bypass surgery performed
- The length of the common, Roux and biliopancreatic intestinal limbs, and size of anastomoses
- The presence of a gastric remnant
- The nature of anastomoses (e.g., with a single anastomosis gastric bypass, is the gastrojejunostomy antiperistaltic or isoperistaltic and the presence of a Braun entero-enterostomy; are anastomosis stapled or handsewn)
- Location of the Roux limb being either retrocolic or antecolic
- Any post-operative surgical complications requiring endoscopic (e.g., any dilation of strictures) or surgical intervention (e.g., marginal ulcer perforation repair)
- Any revisional bariatric procedures – e.g., a patient with sleeve-to-bypass revision will have a smaller gastric remnant making transgastric endoscopic options challenging
- Any other abdominal surgery but most relevant include gallbladder and biliary tree surgery; internal hernia repair; abdominal wall hernia repair (with or without mesh); peptic ulcer surgery
- Previous endoscopy including previous ERCP and sphincterotomy

### 2.4 Preoperative imaging

#### 2.4.1 Cross-sectional imaging

The initial work-up for any periampullary lesion requires high quality dedicated imaging to understand disease stage with respect to primary tumour, lymph node metastases and distant metastases. This will typically involve dedicated CT, MRI and PET imaging, as per local institutional practices. However, the imaging has an added role to assess and confirm the nature of bariatric surgery. The presence of obstruction of the gastric remnant needs to be evaluated – obstruction proximal to the ampulla will affect the success of anterograde approaches to the ampulla (e.g., transgastric access), whereas obstruction beyond the ampulla will affect the success of a retrograde approach (e.g., enteroscopy). However this can be difficult to evaluate as oral contrast will not fill the biliopancreatic limb. Imaging should be reviewed by radiologists experienced in abdominal imaging, as altered post-bariatric anatomy can be difficult to interpret. Beyond the reconstructed anatomy, if patients have had complications from their prior operation, the scarring and fibrosis can make identification and disease extent difficult on CT and MRI, and any regions of chronic inflammation may be avid on PET imaging.

#### 2.4.2 Endoscopic ultrasound

Diagnostic endoscopic ultrasound (EUS) after bariatric surgery is challenging owing primarily to the altered upper gastrointestinal anatomy and access routes. Exclusion of the native stomach and duodenum associated with bypass procedures often prevents standard echoendoscope passage into the usual locations for visualising the pancreas, biliary tree and periampullary region, leading to blind spots and incomplete examinations. The long, angulated limbs of reconstructed small bowel can impede scope advancement and torque, reduce manoeuvrability, and distort anatomical orientation, increasing procedural difficulty with the concern for perforation. As a result, specialised techniques (e.g., device-assisted enteroscopy, transgastric approaches or forward-viewing echoendoscopes) may be required but entail additional expertise.

Studies looking at diagnostic biliary and pancreatic EUS after bariatric surgery are limited to small observational series. Sleeve gastrectomy appears to not impair visualisation of the entire pancreas and biliary tree, however bypass procedures have reduced success rates. Wilson et al. showed that adequate imaging of the head of pancreas and biliary tract with EUS could only be obtained in 6 out of 25 patients who had had a Roux-en-Y gastric bypass (4). Similarly Brozzi et al. had successful imaging in only 5 out of 8 patients after Roux-en-Y gastric bypass; with successful imaging in all 14 patients who had had a sleeve gastrectomy (5).

### 2.5 Pancreaticobiliary access for decompression and biopsy

Biliary decompression and histopathological confirmation of biliary malignancy often requires endoscopic access to the biliary tree. This may be limited by prior bariatric intervention altering upper gastrointestinal anatomy. Procedures such as single anastomosis gastric bypass (SAGB), roux-en-y gastric bypass (RYGB), single-anastomosis duodeno-ileal (SADI) bypass and biliopancreatic diversion with duodenal switch all increase the distance for endoscopic access to the ampulla, often making it impossible without specialised equipment and expertise. This makes understanding patients altered surgical anatomy vital. Endoscopic retrograde cholangiopancreatography (ERCP) and endoscopic ultrasound (EUS) guided biopsy that are the mainstays of biliary assessment, decompression and pancreaticobiliary biopsy are dependent on limited distances from the oropharynx with standard endoscopes. A standard duodenoscope is 124 cm long, and thus distances greater than this may require the use of colonoscopes (168 cm) or enteroscopes (200 cm) for ERCP, adding additional complexity to cannulation due to altered angulation, lack of instrument length for longer endoscope channels, and looping limiting control required for precise control needed for biliary cannulation.

Given the difficulty of obtaining a periampullary biopsy after prior bariatric bypass surgery, imaging therefore becomes critical in guiding management in these patients. Those with a suspicious lesion with clearly resectable disease on imaging may proceed to upfront surgical resection without tissue diagnosis following multidisciplinary review. Conversely, in borderline-resectable or locally advanced disease where neoadjuvant therapies are being considered, biopsy is typically required to establish a histological diagnosis and tailor systemic therapies. The feasibility and choice of endoscopic diagnostic approaches are dictated by the post-bariatric anatomy, specifically whether the duodenum has been excluded and whether a gastric remnant remains accessible.

#### 2.5.1 Non-bypassed bariatric surgery (e.g., sleeve gastrectomy; gastric band; vertical gastroplasty)

Those with a gastric band should have the band deflated to prevent ongoing restriction with regards to nutrition. They must also have a gastroscopy performed to assess if the band has eroded into the stomach. The gastric band should be removed at the time resection, and a missed erosion can lead to a leak or fistula. With these bariatric procedures, the stomach remains in continuity with the duodenum, so ERCP can be performed with a standard duodenoscope with conventional wires and devices. After sleeve gastrectomy, the ability to position the duodenoscope in the long position is restricted, limiting some manoeuvrability, however success rates are equivalent to those without having had bariatric surgery (6).

#### 2.5.2 Bypassed patient in those patients WITH an adequate gastric remnant

With certain bypass procedures, the stomach is divided, with the distal stomach, duodenum and a variable length of proximal jejunum being bypassed, with the gastric remnant remaining in-situ. The most common procedures are the Roux-En-Y gastric bypass and single anastomosis gastric bypass. In these situations, the endoscopic approach to the ampulla can be through accessing the biliopancreatic limb in a retrograde manner through enteroscopy or through approaches that require access to the remnant stomach either laparoscopic, via EUS or percutaneously, allowing anterograde access to the ampulla. If these endoscopic approaches to reach the ampulla fail, than transhepatic approaches have to considered.

##### 2.5.2.1 Enteroscopic assisted ERCP (EA-ERCP)

Enteroscopy assisted ERCP allows access to the ampulla in a retrograde manner, and is an option for those with or without the gastric remnant. It is considered the most minimally invasive method, as it does not involve a surgical perforation to gain access into the gastrointestinal tract, so is typically used as the first option if the expertise is available. Device-assisted enteroscopy techniques (single- or double-balloon, or spiral enteroscopy) allow the Roux limb to be traversed and the excluded duodenum to be reached in a retrograde manner by pleating the small bowel over the scope, effectively “shortening” the intestine and facilitating advancement to the excluded duodenum and papilla. Reaching the ampulla in this manner is particularly challenging when the combined roux and biliopancreatic limbs exceed 150 cm (7). Cannulation is complicated by the forward-viewing enteroscope and absence of an elevator. As the ampulla is approached retrograde, the bile duct usually lies at the 5–6 o'clock position, rather than the conventional 11–12 o'clock position. Even after cannulation, the longer length of the enteroscope (200 cm) and a narrow working channel (2.8 mm), restrict what devices can be used through the scope including the compatibility of certain stents. For these reasons, reported overall technical success rates for enteroscopy-assisted ERCP in this setting are typically around 60%–70% (8), lower than transgastric approaches, and procedures are technically demanding with longer procedure times (9).

##### 2.5.2.2 Transgastric approaches

Transgastric approaches rely on access to the excluded gastric remnant to permit anterograde duodenal intubation with a standard side-viewing duodenoscope, analogous to conventional ERCP. Such access may be achieved via laparoscopic-assisted ERCP (LA-ERCP), EUS-Directed transGastric ERCP (EDGE), or percutaneous transgastric techniques. However, when malignant disease also results in obstruction of the gastric remnant, these transgastric strategies may not be feasible if the obstruction is proximal or involving the ampulla. In patients with normal gastrointestinal anatomy, gastric outlet obstruction is often predicted clinically by intolerance of oral intake, nausea, or vomiting; in contrast, patients with bariatric bypass will not display these symptoms. Consequently, careful pre-operative scrutiny of cross-sectional imaging is essential to determine whether the gastric remnant is obstructed and to assess the likelihood of successful transgastric access. Radiological features suggestive of gastric outlet obstruction include mural thickening of the first and/or second portions of the duodenum with a defined transition point, and distension of the gastric remnant. When such findings are present, than the clinician should anticipate alternative approaches may be necessary with a clear patient counselling prior to a transgastric attempt.

###### 2.5.2.2.1 Laparoscopic assisted ERCP (LA-ERCP)

In the laparoscopic-assisted ERCP (LA-ERCP) approach, the abdomen is accessed laparoscopically and the excluded gastric remnant is identified. A 15–18 mm port is placed directly into the gastric lumen under vision and secured to the abdominal wall with seromuscular sutures to create a controlled gastrostomy. The port needs to be at least 15 mm to accommodate a standard therapeutic duodenoscope. To optimize scope manoeuvrability and facilitate duodenoscopy, the gastrostomy is ideally positioned on the anterior gastric body as far from the pylorus as anatomy permits (10), and the pneumoperitoneum is released during endoscope passage to reduce gastric wall tension. Use of a conventional duodenoscope allows deployment of the full range of standard ERCP accessories, including large-diameter stents including self-expanding metal stents (SEMS). After completion of the endoscopic intervention, the duodenoscope is withdrawn, the gastric port removed, and the gastrostomy laparoscopically closed. In situations where repeat biliary access is anticipated (e.g., concern for post-sphincterotomy bleeding; unclear diagnosis requiring repeated ERCP and/or EUS; concern about adequacy of biliary drainage), a large-diameter gastrostomy tube may be left *in situ* to maintain access. Reported technical and therapeutic success rates for LA-ERCP exceed 90%–95% in experienced centres (11). Potential morbidity includes gastrotomy-related complications such as leak or abscess, risk of conversion to laparotomy (7%), and standard ERCP-related adverse events including pancreatitis, bleeding, and stent migration or occlusion (11). Post-operative adhesions from previous abdominal surgery can make this procedure challenging.

###### 2.5.2.2.2 EUS directed transGastric ERCP (EDGE)

In the EUS-directed transgastric ERCP (EDGE) approach, the excluded gastric remnant is accessed using endoscopic ultrasound from the gastric pouch (or from the proximal jejunum when the pouch is small). Under EUS guidance, the gastric remnant is identified and punctured with a fine-needle aspiration needle; correct positioning is confirmed by contrast injection under fluoroscopy (9, 10, 12). Careful assessment for intervening vessels and adjacent viscera is essential prior to attempting access. The remnant stomach is then distended with fluid and a lumen-apposing metal stent (LAMS) is deployed, creating a gastro-gastrostomy tract between the pouch and the excluded remnant. This conduit permits passage of a standard duodenoscope to the ampulla in the usual anterograde fashion, allowing ERCP to be performed in a conventional manner. The presence of a hiatus hernia will make EDGE incredibly difficult, if not impossible depending on the size and anatomy of the hernia.

Traditional EDGE protocols recommended a 3–4 week interval between LAMS placement and duodenoscopy to permit fistula maturation, based on concern that immediate duodenoscopy might precipitate LAMS migration and free perforation. However, such delays are often impractical in the setting of malignant biliary obstruction, particularly in patients with acute cholangitis or symptomatic jaundice. To improve the safety of single session EDGE, strategies employed to reduce the risk of stent migration including using a larger calibre stent, balloon dilation and stent fixation (12, 13). Recent meta-analyses report technical success rates of approximately 90%–100% (12), with single-session EDGE associated with LAMS migration rates in the order of 8%–9% (13). The LAMS is typically removed after 4 weeks with primary closure of the gastro-gastrostomy, although the LAMS may be left *in situ* when repeat endoscopic access is anticipated. A key feature is that the LAMS in essence “reverses the bypass”, allowing food to go through the conventional pathway into the duodenum and enter the bypassed circuit. This could allow for weight gain along with nutritional benefits in the pre-operative period. Other specific complications of EDGE include gastric content leakage from the gastrotomy, bleeding from the gastro-gastric fistula and persistence of the gastro-gastric fistula, the latter of which warrants particular vigilance at the time of oncologic resection, all of which occur at a rate of 15%–30% (12). As EDGE is an entirely endoscopic intervention, it avoids the challenges posed by postoperative adhesions that may limit feasibility of LA-ERCP. As EDGE can be done under sedation (14), this procedure becomes an option for patients who are unable to tolerate a general anaesthetic.

###### 2.5.2.2.3 Percutaneous access to gastric remnant

Percutaneous approaches to the gastric remnant are well described in the context of gastrostomy tube placement. Conventional techniques typically rely on gastric insufflation via a nasogastric tube to enhance safety during gastric puncture; however, this is not feasible in the bypassed patient. Nonetheless, percutaneous access to the gastric remnant may still be achieved under CT, fluoroscopic, or ultrasound guidance (15, 16), followed by serial tract dilatation to permit placement of an appropriately sized access port and subsequent duodenoscope passage, similar to LA-ERCP (17). In the absence of laparoscopic abdominal access, the gastrostomy cannot be closed at the conclusion of the procedure, and a gastrostomy tube must therefore be left *in situ* to prevent free perforation.

Device-assisted enteroscopy or endoscopic ultrasound can also facilitate percutaneous gastric remnant access (9). During enteroscopy, if the afferent limb is reached but biliary cannulation is unsuccessful, or if required accessories are unable to navigate the scope, the enteroscope can be advanced into the gastric remnant to guide percutaneous puncture (18). A separate approach uses EUS, to visualise the gastric remnant, enabling percutaneous access through the abdominal wall (19). This EUS-guided approach may be particularly useful when an EDGE procedure is being attempted but transluminal access through the pouch is challenging or unsafe, for example due to intervening viscera or blood vessels.

Overall, percutaneous transgastric approaches to facilitate ERCP after bypass surgery are far less frequently reported, with contemporary literature focusing predominantly on EA-ERCP, LA-ERCP, and EDGE techniques. The technical difficulty of accessing and dilating the gastric remnant tract, coupled with the obligatory placement of a gastrostomy tube post-procedure, likely contributes to the lower utilisation of the percutaneous approach. Gangwani et al. (7) recently performed a network meta-analysis to compare the comparing EA-ERCP, LA-ERCP and EDGE. The technical success rates observed was 94.8% with EDGE; 95.0% with LA-ERCP, and 66.3% with EA-ERCP; with EA-ERCP being statistically different from the others. The adverse event rates, was 14.9% with EDGE; 20.3% with LA-ERCP and 14.7% with EA-ERCP, with each not being statistically different from the others. When comparing procedural time, EDGE was 31 min shorter than EA-ERCP, and 78 min shorter than LA-ERCP.

#### 2.5.3 Those patients WITHOUT an adequate gastric remnant

##### 2.5.3.1 Endoscopic

Patients may present either without a gastric remnant or with a markedly reduced remnant stomach. In duodenal switch procedures, the sleeved stomach is divided at the first part of the duodenum and then transposed to the distal small intestine, preventing any transgastric access to the ampulla. With patients having a Roux-en-Y, some patients may have had the gastric remnant resected, particularly in those at increased risk of gastric malignancy or with distal gastric pathology likely to cause ongoing problems. Endoscopic intervention can be achieved using enteroscopy-assisted ERCP (EA-ERCP) as described above, or by laparoscopic-assisted ERCP, with access obtained via a transjejunal enterotomy, with then retrograde access to the ampulla (20). The enterotomy is ideally fashioned on the biliopancreatic limb to minimise the distance to the ampulla and to permit the use of a standard side-viewing duodenoscope rather than an enteroscope (21).

Patients undergoing conversion from sleeve gastrectomy to gastric bypass typically have a small, tubular gastric remnant, which renders transgastric approaches technically challenging. Smaller gastric remnants are harder to access via EUS, radiologically or laparoscopically due to their smaller size along with their location being under the left lobe of the liver. Furthermore the access trocars are limited to being placed closer to the pylorus constraining endoscope manoeuvrability during ERCP. The surgical team must consider the original indications for revision, as conversions performed for complications such as sleeve leaks can be associated with dense adhesions that may make identification of the remnant during laparoscopy difficult or unsafe. Isolated cases of EUS-directed transgastric ERCP (EDGE) in this setting have been reported (22, 23), however, endoscopic access is more commonly achieved with EA-ERCP, with a transjejunal enterotomy–assisted ERCP also an additional option.

### 2.6 Transhepatic approaches

ERCP may fail even in patients with normal gastrointestinal anatomy, owing to patient-specific anatomical factors (for example, an ampulla located within a deep duodenal diverticulum) or pathology-related issues such as gastric outlet obstruction that preclude endoscopic access to the duodenum. In such settings, percutaneous transhepatic biliary intervention, performed by interventional radiological services, is a well-established alternative for biliary decompression and intervention. These techniques can also be applied to patients with altered anatomy after bariatric surgery (24).

Percutaneous transhepatic biliary access is obtained by puncturing a peripheral intrahepatic duct under ultrasound and/or fluoroscopic guidance, followed by antegrade catheterisation of the common hepatic duct and common bile duct, with the option of crossing the ampulla into the duodenum. Procedural success is facilitated with a dilated biliary system, but will still often be successful in a non-dilated biliary tree. After access is secured, antegrade cholangiography, drainage, duct clearance, and stent placement can be performed. Percutaneous transhepatic cholangioscopy will allow for direct visualisation, targeted biopsies, and therapy (25). These interventions are typically undertaken in a staged fashion, beginning with establishment of the percutaneous tract, followed by definitive biliary intervention. Once no further biliary access is required, the biliary drain is removed and the liver tract embolised to minimise the risk of bile leak. Transhepatic approaches have also been described via EUS access through the gastric pouch (10).

### 2.7 Pre-operative stenting

If a pre-operative biopsy is being considered, than the treating team must think critically with regards to pre-operative stenting as repeated access to the ampulla and biliary tree may not be straightforward. This can make it difficult to remove, clear or exchange stents. In patients who undergo resection, pre-operative stenting is also associated with increased risk of complications including cholangitis and wound infection (26, 27). Current NCCCN and ESMO guidelines generally recommend against routine preoperative stenting for resectable periampullary tumours, unless there are specific clinical reasons such as (28, 29):

- Acute cholangitis
- Severe symptomatic jaundice
- Delays before planned surgery e.g., neoadjuvant therapy
- Markedly elevated bilirubin (typically bilirubin >250micromol/L)

Plastic biliary stents are inexpensive and easy to insert, and importantly they induce less tissue reaction than metal stents; this can be advantageous when diagnostic uncertainty remains, as the lower degree of hyperplastic or inflammatory ingrowth preserves accuracy for repeated tissue biopsies (30). However, plastic stents have smaller diameters, higher occlusion rates, and typically require frequent exchanges—an important drawback in post-bypass anatomy. Self-expanding metal stents (SEMS) provide larger luminal calibre, longer patency, and fewer interventions (31, 32). However the expanding nature of the SEMS, can lead to pancreatic duct obstruction, precipitating pancreatitis (33). Uncovered SEMS allow for greater tissue ingrowth with prevents the stent from migrating, making them better suited for unresectable disease where durable palliation is the goal (34). Fully covered SEMS are removable, but with less tissue ingrowth, they have a higher risk of migration (35), particularly if a patient responds well after neoadjuvant therapies. In a patient with a gallbladder *in situ*, a fully covered SEMS will occlude cystic duct which can precipitate cholecystitis (36). Some surgeons have believed that metal stents induce higher tissue reaction around the biliary tree which can complicate resection but this has not been consistently shown in the literature (31). Decisions regarding pre-operative stenting relies on careful staging to determine resectability: cross-sectional imaging, EUS, and MDT discussion are central to defining vascular involvement, metastatic spread, and surgical feasibility (37). When disease is upfront resectable —or when diagnostic uncertainty persists—plastic stenting or a fully covered SEMS may be preferred as these can be removed. Conversely, in definitively unresectable disease, an uncovered SEMS generally offers the best long-term biliary drainage with minimal need for repeat intervention. In those requiring neoadjuvant chemotherapy, maintaining stent patency is key to minimise re-intervention, infective complications and time off therapy. The choice of an uncovered or covered metal stent becomes matter of surgeon/institution preference. Seo et al. reported that although both covered and uncovered stents provide similar rates in treating biliary obstruction; the reasons for failure with uncovered stents was tumour ingrowth, whereas with covered stents the failure was due to stent migration (38). As such, repeat intervention is still not infrequently required, so utilising an EDGE technique allows leaving a permanent LAMS behind for repeated access to the ampulla. This may also have the added benefit of reversing the bypassed anatomy allowing for improved weight and nutrition. If a LA-ERCP in being performed, than a gastrostomy tube can be left *in-situ* allowing for repeat endoscopy, without requiring repeat laparoscopy, whilst also providing access for supplemental enteral nutrition.

## 3 Operative considerations

The presence of a previous bariatric procedure adds additional complexity during the time of surgical resection. It becomes key that the surgical team understand the patient's anatomy and pathology. The decisions on whether completion gastrectomy should be performed, route of pancreaticobiliary drainage and what to do with the original Roux limb are subject to debate (39–41).

### 3.1 Non-bypassed bariatric surgery

#### 3.1.1 Sleeve

After sleeve gastrectomy, that dissection into the lesser sac may challenging due to post-operative adhesions. The left and right gastroepiploic vessels have been ligated during creation of a sleeved stomach along with the short gastric vessels, with the stomach being supplied by the left and right gastric vessels. Nevertheless, some advocate preserving the right gastric vessels at the time of resection to minimise gastric ischaemia 41.

#### 3.1.2 Gastric band and vertical band gastroplasty

With those who have had a gastric band, these should be removed as they can become a nidus for post-operative infection. If not already done so, the gastric band should be deflated prior to surgery. The band is typically placed on the proximal stomach, typically surrounded by dense adhesions. Once the band has been removed, the upper stomach should be left alone, with the gastrojejunostomy done on a region of the stomach well away from this area. If a full thickness band erosion is pre-operatively known, than the band can be removed through a gastrotomy made well away from the band (most easily done at the time of stomach transection), with the band removed from within the stomach, leaving the dense capsule around the band intact. Otherwise a partial gastrectomy may be required. Similarly a partial or subtotal gastrectomy has to be considered in those who have had a previous vertical band gastroplasty (41).

### 3.2 Bypassed bariatric surgery

Before undertaking extensive dissection, the surgeon should carefully delineate the post-bypass anatomy, confirming the configuration of intestinal limbs, anastomoses, and the presence or absence of a gastric remnant. This assessment may be complicated by postoperative adhesions. Accurate definition of the anatomy directly informs subsequent decisions regarding both resection and reconstruction.

#### 3.2.1 During dissection and resection of the specimen

Dissection of the specimen should be performed in usual manner as is for pancreaticoduodenectomy, without compromising any oncological margins or lymph node stations. Challenges during resection created by pre-existing bypass surgery that the surgeon should be watchful for include:

- **Hostile left upper quadrant,** due to post-operative adhesions, or from post-operative complications (e.g., anastomotic leak) (39).
- **Location of the Roux-limb**. During entry into the lesser sac and dissection onto the superior mesenteric vein (SMV) and superior mesenteric artery (SMA), the roux limb will be seen traversing to the gastric pouch either in a retrocolic or antecolic position. It is in this retrocolic position that the Roux limb and its mesentery is most likely to be injured as it traverses through the lesser sac, and posterior adhesions may make exposure of the SMV/SMA pedicle difficult. If in the retrocolic position, it is most commonly positioned on the left of the middle colic pedicle.
- **Orientation of jejunal mesentery.** Mobilisation of the jejunum to construct the Roux limb can alter the orientation of jejunal arcades, course and branching pattern of the SMA, and venous drainage pathways into the SMV, making it difficult to identify the first jejunal branch of the SMV, the inferior-pancreaticoduodenal vessels and the SMA-SMV relationship during uncinate dissection.
- **Abnormal vascular anatomy.** Prior surgery and construction of intestinal limbs can create focal areas of increased mesenteric (portal) venous pressures driving collateral neovascularisation within the mesentery, with friable vessels crossing adhesions and mesenteric planes. The bleeding from these “hidden bleeders” can be substantial which can retract complicating dissection.
- **Gastric remnant.** A key decision step is determine whether the gastric remnant should be resected or left in-situ. Removing the gastric remnant avoids creating an extra anastomosis that could either bleed or leak in the acute scenario, or develop marginal ulcers or stricture in the future. Any endoscopic therapy to this anastomosis will also be difficult as it is excluded from the main gastrointestinal passage. Considering the higher density G-cells in the antrum, removing the gastric remnant may also lead to reduced marginal ulcers by lowering acid production at the gastric pouch. Resection of the remnant also avoids the risk of a gastric remnant malignancy. If the gastric remnant is removed, surgeons should be cautious with adhesions after any transgastric endoscopic procedures and particularly look carefully for any gastro-gastric fistulas with the gastric pouch. However removing the remnant will require considerable extra dissection, with risks of injury to the gastric pouch or Roux limb. The gastric remnant can also provide access for gastrostomy tubes for supplemental nutrition if required, and for creation of a pancreaticogastrostomy (42). Most described case series/reports have resected the gastric remnant (43). If the gastric remnant is removed, care has to be taken not to injure the left gastric artery, as this becomes the primary blood supply to the gastric pouch, though there can be extra contribution from oesophageal branches through the oesophageal hiatus or from short gastric arteries.

#### 3.2.2 Route of reconstruction

Multiple reconstructive strategies following pancreaticoduodenectomy in patients with prior gastric bypass have been described (40, 41, 44). The key decision step is to determine whether sufficient length of the existing biliopancreatic limb remains after resection to permit safe, tension-free reconstruction. Key principles guiding reconstruction include:

1. Appropriate drainage of any retained gastric remnant.

If a gastric remnant is preserved, it must be adequately drained, either via the biliopancreatic limb or through construction of a new limb. The Roux limb or the gastric pouch should be avoided for this purpose, as it risks entry of food into the remnant with subsequent stasis and its complications.

(1) Correct sequencing of alimentary flow relative to biliary and pancreatic anastomoses.

Where sufficient biliopancreatic limb length is available, this limb is preferred to be used to construct the pancreaticojejunostomy (PJ), hepaticojejunostomy (HJ), and, where required, drainage of the gastric remnant (40, 41). The limb is typically divided just distal to the ligament of Treitz with the residual limb mobilised in a retrocolic fashion as is usual with reconstruction. An approximate length of 50 cm of residual limb should be endeavoured to allow for tension free anastomosis at the PJ and HJ. If the residual biliopancreatic limb is inadequate, the surgeon needs to decide on whether it should be resected completely back to the jejunojejunostomy to avoid blind loop formation and bacterial overgrowth; or if it can be used to drain the gastric remanent in isolation. Reconstruction then requires creation of a new biliopancreatic limb for the pancreatic and biliary anastomoses either by using the common channel or the Roux limb, and then formation of a new jejunojejunostomy. If using the Roux limb, it must be divided as the gastric pouch should not be placed in series proximal to the biliary and pancreatic anastomosis so to prevent foodstuffs occluding the pancreatic duct and bile duct causing pancreatitis and cholangitis respectively (40).

(1) Preservation of an adequate intestinal limbs to minimise the risk of clinically significant malabsorption or short gut syndrome.

The amount of bypassed intestine may need to be reduced, particularly in those who have had a previous duodenal switch procedures, where the length of the common channel may well be <100 cm. In this scenario, there will be ample length of biliopancreatic limb to create the HJ and PJ, and the surgeon should make an assessment to see if any biliopancreatic limb should be re-introduced into the Roux-limb to reduce the amount of bypassed intestine to improve post-operative nutrition.

Multiple variations are described in the literature relating to individual anatomical and disease factors. The post-operative note should describe the anatomy well including location and orientation of anastomoses, and the length of intestinal limbs including the length of the common channel. This is best done with a diagram.

#### 3.2.3 Unresectable/metastatic disease

On entry, the abdomen should be assessed for metastatic or unresectable disease before irreversible surgical steps. If unresectable, palliative strategies should be considered. In cases of gastric remnant outlet obstruction, a bypass anastomosis between the gastric remnant and jejunum should be considered to prevent perforation of the gastric remnant. Biliary drainage can be addressed via surgical bypass)—hepatico-jejunostomy (HJ) or choledocho-duodenostomy (CD)—or on-table ERCP through transgastric or transjejunal access. When performing a HJ, intestinal limbs should be carefully traced, with preference for the biliopancreatic limb as this is most physiological; the Roux limb should be avoided to prevent cholangitis from foodstuff occluding the biliary anastomosis; the common channel should be avoided to reduce the risk of bile acid diarrhoea. The biliopancreatic limb may have to be divided to facilitate the limb to reach, with the distal end of the intestinal limb being used to anastomose to the bile duct, and the proximal end anastomosed to the Roux limb or common channel. CD may be preferred if adhesions or peritoneal disease limit jejunal mobility, however the tumour must not be involving the second or third parts of the duodenum, nor must the tumour be heading into the proximal biliary tree (45, 46). A key limitation of on-table endoscopic approaches is that, should the stent occlude or migrate, repeat endoscopic access may be technically challenging or impossible in the altered post-bypass anatomy. As repeated endoscopic access is difficult, if unresectable is found, surgical biliary bypass should strongly be considered even if the patient does not currently have a biliary obstruction.

## 4 Post operative considerations

Optimisation of nutrition is critical following pancreaticoduodenectomy, as postoperative malnutrition is associated with increased surgical morbidity and delays in the initiation of adjuvant oncological therapies (47). These nutritional challenges can be compounded by their original bariatric procedure if there are still significant lengths of the gastrointestinal tract being bypassed. There is lack of literature of the specific post-operative care requirements of those who have had bariatric surgery who then undergo a pancreaticoduodenectomy. However, general nutritional challenges after pancreaticoduodenectomy include (48):

- delayed gastric emptying (DGE), which can be managed with prokinetics such as erythromycin or metoclopramide; but in severe cases requiring jejunal feeding tubes or parental nutrition. In those with previous gastric bypass surgery, the development of DGE is unpredictable (39, 40).
- neurogenic diarrhoea from arterial divestment, requiring loperamide (49)
- steatorrhea due to inadequate exocrine pancreatic function, requiring pancreatic enzyme replacement
- poor glycaemic control
- small intestinal bacterial overgrowth from any blind intestinal loops
- deficiencies in trace elements

These nutritional complications are often unpredictable post-surgery, made more challenging to manage in the context of the original bariatric procedure. Nutritional treatment decisions are nuanced, and should be managed through a multidisciplinary team involving dieticians, pharmacists and endocrinologists, particularly as enhance recovery after surgery (ERAS) pathways are increasingly being adopted.

## 5 Discussion

The altered anatomy and physiology following bariatric surgery introduce significant complexity in the assessment and management of pancreaticobiliary malignancies. Diagnostic evaluation, endoscopic access, and operative strategy may all be substantially affected by the type of prior bariatric procedure, necessitating individualized medical and surgical approaches. This will continue to be a growing problem as those patients who have had their bariatric surgery 10–20 years ago, will now start entering the age bracket where pancreaticobiliary malignancies arise. Decisions are not straightforward and rely upon good coordination between the multidisciplinary team to tailor the patient's management. This review provides a framework for treating clinicians when faced with such clinical challenges.
